# Engineering better biomass-degrading ability into a GH11 xylanase using a directed evolution strategy

**DOI:** 10.1186/1754-6834-5-3

**Published:** 2012-01-13

**Authors:** Letian Song, Béatrice Siguier, Claire Dumon, Sophie Bozonnet, Michael J O'Donohue

**Affiliations:** 1 Université de Toulouse; INSA, UPS, INP; LISBP, 135 Avenue de Rangueil, F-31077 Toulouse, France; 2 INRA, UMR792, F-31400 Toulouse, France; 3 CNRS, UMR5504, F-31400 Toulouse, France; 4 CNRS, Institut de Pharmacologie et de Biologie Structurale, F-31077 Toulouse, France

**Keywords:** Directed evolution, high-throughput screening, endo-β-1,4-xylanase, lignocellulosic biomass, synergistic interaction, biorefining

## Abstract

**Background:**

Improving the hydrolytic performance of hemicellulases on lignocellulosic biomass is of considerable importance for second-generation biorefining. To address this problem, and also to gain greater understanding of structure-function relationships, especially related to xylanase action on complex biomass, we have implemented a combinatorial strategy to engineer the GH11 xylanase from *Thermobacillus xylanilyticus *(Tx-Xyn).

**Results:**

Following *in vitro *enzyme evolution and screening on wheat straw, nine best-performing clones were identified, which display mutations at positions 3, 6, 27 and 111. All of these mutants showed increased hydrolytic activity on wheat straw, and solubilized arabinoxylans that were not modified by the parental enzyme. The most active mutants, S27T and Y111T, increased the solubilization of arabinoxylans from depleted wheat straw 2.3-fold and 2.1-fold, respectively, in comparison to the wild-type enzyme. In addition, five mutants, S27T, Y111H, Y111S, Y111T and S27T-Y111H increased total hemicellulose conversion of intact wheat straw from 16.7%_tot. xyl _(wild-type Tx-Xyn) to 18.6% to 20.4%_tot. xyl_. Also, all five mutant enzymes exhibited a better ability to act in synergy with a cellulase cocktail (Accellerase 1500), thus procuring increases in overall wheat straw hydrolysis.

**Conclusions:**

Analysis of the results allows us to hypothesize that the increased hydrolytic ability of the mutants is linked to (i) improved ligand binding in a putative secondary binding site, (ii) the diminution of surface hydrophobicity, and/or (iii) the modification of thumb flexibility, induced by mutations at position 111. Nevertheless, the relatively modest improvements that were observed also underline the fact that enzyme engineering alone cannot overcome the limits imposed by the complex organization of the plant cell wall and the lignin barrier.

## Background

Wheat straw is an abundant coproduct of the agri-food industry that is currently considered to be a primary source of lignocellulosic biomass for second-generation biorefining. The composition of wheat straw is typical of graminaceous species, containing arabinoxylan (20% to 25% dry weight (DW)), cellulose (35% to 45% DW) and lignins (15% to 20% DW) in variable proportions that are determined by both cultivar characteristics and pedoclimatic differences [1,2]. Regarding the ultrastructure of wheat straw, the internode regions, which in DW terms represent the majority of wheat straw, are characterized by different tissue types, which notably display different levels of lignification. The central cavity, or lumen, of straw is lined by pith that covers parenchyma cells and that possesses mainly primary cell walls. Moving further outwards to the external part of wheat straw, one can identify sclerenchyma cells, xylem tissue and finally the outer epidermis, all of which possess lignified secondary cell walls [3,4].

Endo-β-1,4-xylanases (EC 3.2.1.8, xylanase) randomly depolymerize the backbone of β-1,4-linked xylans [5], including arabinoxylans such as those found in wheat straw. Current commercial uses for xylanases mainly focus on the paper, food and animal feed industries [6,7], but it is increasingly recognized that these will also be important for biorefining of lignocellulosic biomass [8,9]. Indeed, recent studies have shown that xylanases are needed in cellulase cocktails in order to alleviate the inhibition of various cellulose-degrading enzymes by xylo-oligosaccharides [10]. Also, the development of ambitious approaches such as consolidated bioprocesses [11], which require the use of microorganisms possessing the dual ability to degrade complex biomass and convert the fermentable sugars into useful products, will also create new demands for highly efficient xylanolytic systems.

**Figure 1:**
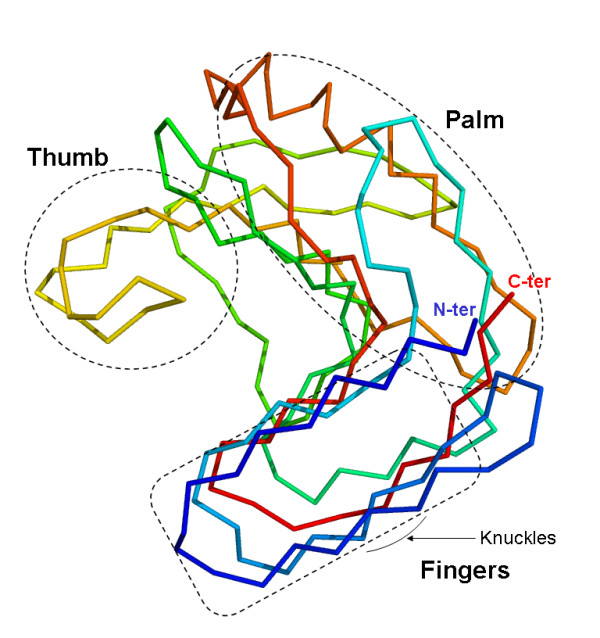
**Ribbon representation of *Thermobacillus xylanilyticus *xylanase (Tx-Xyn) three-dimensional structure**. The schematic protein is 'color-ramped' from the N-terminus (blue, N-ter) to the C-terminus (red, C-ter). The relevant regions of 'thumb', 'palm' and 'fingers' are highlighted in frames, and the 'knuckles' in the fingers region are indicated by an arrow.

To date, most industrial processes that employ xylanases use enzymes that belong to the glycoside hydrolase family GH11 [12]. Bacterial GH11 xylanases are mostly single domain enzymes that exclusively act on β-1,4 links between xylosyl units in xylans and display a β-jelly roll structure that has been likened to a partially folded human right hand (Figure 1) [13]. Likewise, the prominent elements of the GH11 three-dimensional structure, which is composed mainly of two β sheets and one α helix, have been identified using terms such as 'thumb', which describes a large mobile loop that is located above the active site cleft, 'palm', whose half-folded structure forms the active site cleft, and fingers, which constitute one side of the active site cleft and whose 'knuckles' bear a secondary substrate binding motif [14,15].

Despite the fact that xylanases will be necessary for biorefining operations, very little R&D has so far been focused on the improvement of xylanases specifically for biorefining purposes, and in particular for increased activity on complex biomass. This is partly because a lot of effort has been focused on cellulase engineering, and also because presently it is unclear on what basis improvements could be achieved. Regarding the action of xylanases on lignocellulosic biomass that has not been subjected to prior pretreatment, very little is known, though some studies of GH11 xylanase from *Thermobacillus xylanilyticus *(designated Tx-Xyn) actions on wheat bran and straw, and have provided insight into the factors that might determine overall enzyme efficiency. Nevertheless, the available information is still sparse, making the prospect of rational engineering rather haphazard.

Alternatively, random approaches coupled to enzyme *in vitro *evolution could be a suitable way to tackle xylanase engineering. So far, the use of such techniques on xylanases has been limited to the improvement of thermostability [16-20] and alkaliphilicity [21-23]. In these studies, screening methods relied on the use of isolated xylans, such as Remazol Brilliant Blue (RBB)-xylan and birchwood xylan. However, in a recent study we have developed a new microtiter plate-based screening method that is far more suitable for the study of xylanase action on complex biomass [24]. Therefore, in this paper, we describe the use of this screening procedure in an enzyme engineering project that has focused on the moderately thermostable Tx-Xyn. This enzyme was selected, because it has already been extensively studied, notably with regard to its activity on insoluble complex substrates such as wheat bran and straw, which is not the case for other GH11 xylanases [25-28]. Using a combination of random mutagenesis and DNA shuffling, we have isolated several Tx-Xyn variants that showed increased activity on wheat straw and improved synergistic action, when used in combination with a commercial cellulase preparation.

## Results

### Screening of randomly mutagenized xylanase libraries

**Figure 2:**
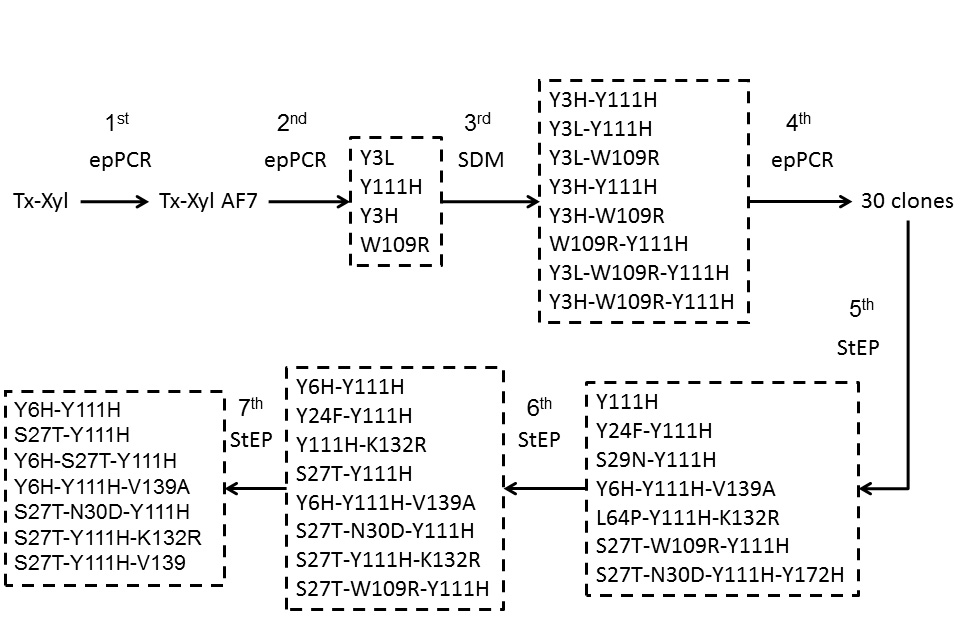
**Flowchart of the *in vitro *evolutional process**. The best-performing mutants, used as parental input for a subsequent round of evolution, are boxed and mutants are designated according to the point mutations that characterize them.

The different steps of the engineering strategy are summarized in Figure 2. The initial phase of this work involved the use of error-prone PCR (epPCR) to generate random biodiversity. In preliminary work, we observed that more than 10 base mutations/kb produced >70% inactive clones. Therefore, a progressive strategy employing three successive rounds of epPCR was preferred, with moderate mutational charge (5 to 7 base mutations/kb) at each stage. The results of activity screening (where activity can generally be considered to be the product of both expression levels and specific activity) at each round are summarized in Table 1. Regarding the first round of screening, this work has already been reported by Song *et al*. [24]. Although the best mutant from this first round, designated Tx-Xyn-AF7, displays a wild-type amino acid sequence, its DNA sequence contains two mutations (at nucleotide positions 27 and 516) that cause approximately twofold higher expression of the recombinant enzyme. Therefore, the sequence encoding Tx-Xyn-AF7 was used as the template for the second round of epPCR.

**Table 1:** Summary of directed evolution for improvement of *Thermobacillus xylanilyticus *xylanase (Tx-Xyn) xylanase activity

Library type	Substrate	Variants screened	CV WT	Percentage of clones with improved activity					No. of hits selected
					
				>4C	>5C	>6C	>7C	>8C	
				V	V	V	V	V	
epPCR	In-WS	264	11.1 ± 1.3%	0.4%	0.4%	-	-	-	1
epPCR	In-WS	4,333	18.1 ± 5.4%	0.1%	-	-	-	-	4
SDM	-	-	-						11
epPCR	Dpl-WS	4,300	10.9 ± 2.2%	1.2%	0.6%	-	-	-	30
Shuffling	Dpl-WS	3,840^a ^(approximat ely 2,500)	8.1 ± 0.6%	1.4%	6.0%	2.1%	0.8%	0.1%	7
Shuffling	Dpl-WS	864^a ^(1,847)	10.2%	9.3%	2.8%	0.9%	0.2	-	8
Shuffling	Dpl-WS	864^a ^(127)	11.3%	19.5%	7.5%	2.4%	0.5%	0.2%	7

CV = coefficient of variation; Dpl-WS = xylanase-depleted wheat straw; epPCR = error-prone PCR; In-WS = intact wheat straw; SDM = site-directed mutagenesis; WT = wild-type.

^a^Values in brackets are the number of theoretical mutational combinations.

DNA sequence analysis of ten library clones, taken from the second-generation library, revealed an average mutation rate of 5.4 base substitutions/kb and a transition/transversion ratio of approximately 1.4, indicating that the mutations were relatively unbiased in this respect. A total of 4,333 clones were screened on intact wheat straw (In-WS), and the 4 most active clones (>4CV) were selected, using the activity of Tx-Xyn-AF7-bearing clones as the base case for comparison. DNA sequencing revealed that all four clones were characterized by single amino acid changes. Two clones were mutated at position 3 (Y3L and Y3H), while two others were mutated at independent, but neighboring locations (W109R and Y111H).

Examination of the three-dimensional structure of Tx-Xyn revealed that Y3 lies in the distal glycon part of the active site cleft, while W109 and Y111 are situated nearby and in the thumb region, respectively; thus all three residues are potentially important for enzyme function. For this reason, at this stage in the experiment it was decided to focus on these mutations for the creation of further mutant libraries. However, to ensure that all of the possible permutations would be present in the third generation, recombination was achieved using site-directed mutagenesis. Consequently, five double mutants (Y3L-W109R, Y3L-Y111H, Y3H-W109R, Y3H-Y111H and W109R-Y111H) and two triple mutants (Y3L-W109R-Y111H and Y3H-W109R-Y111H) were created. Together with the other four original single mutants, these were used as parental templates for the next round of epPCR, which led to the creation of a fourth generation (Figure 2).

To efficiently challenge clones present in the fourth library, the microtiter plate assay was modified by replacing In-WS with xylanase-depleted wheat straw (Dpl-WS). The principle behind this was to select clones that produce enzymes that can actually hydrolyze arabinoxylans that are inaccessible or resistant to wild-type xylanase. The key features and performance descriptors of this modified assay are summarized in Table 2. Overall, the CV value for individual wells of Tx-Xyn-AF7 control varied between 8% to 11%, indicating that this screen was sufficiently reliable for library screening.

**Table 2:** Characteristics of intact wheat straw (In-WS) and xylanase-depleted wheat straw (Dpl-WS) and summary of the two screening assays

	In-WS screening	Dpl-WS screening
Substrate properties:		
Substrate type	Intact wheat straw	Xylanase-depleted wheat straw
Particle size	Average 0.5 mm	
Glucose, % (w/w)	44.51 ± 0.08%	45.69 ± 0.94%
Xylose, % (w/w)	26.16 ± 0.14%	21.92 ± 0.17%
Arabinose, % (w/w)	2.37 ± 0.03%	2.05 ± 0.07%
Ratio of Ara:Xyl	0.091	0.094
Screening conditions:		
Weight (mg per microplate)	420 to 440	385 to 405
Cell-free extract (CFE) loading	CFE in NaOAc,	pH 5.8, 250 μl/well
Temperature and time	60°C, 4 h	60°C, 16 h
Sealing	Aluminum film	Polypropylene film
Evaporation (w/w, %)	1.44 ± 0.16%	0.23 ± 0.05%
Activity assay	Micro-DNS assay	

DNA sequence analysis of a randomly picked sample of fourth-generation library clones revealed an average mutation rate of 7.2 nucleotide substitutions/kb. Likewise, functional screening using the modified Dpl-WS assay indicated that 0.6% of screened clones presented activities that were significantly higher (>5CV) than the mean value of the activity of Tx-Xyn-AF7 clones. Therefore, the top 30 clones were isolated and used for subsequent rounds of DNA recombination.

### Optimization of mutant xylanases using DNA recombination

**Figure 3:**
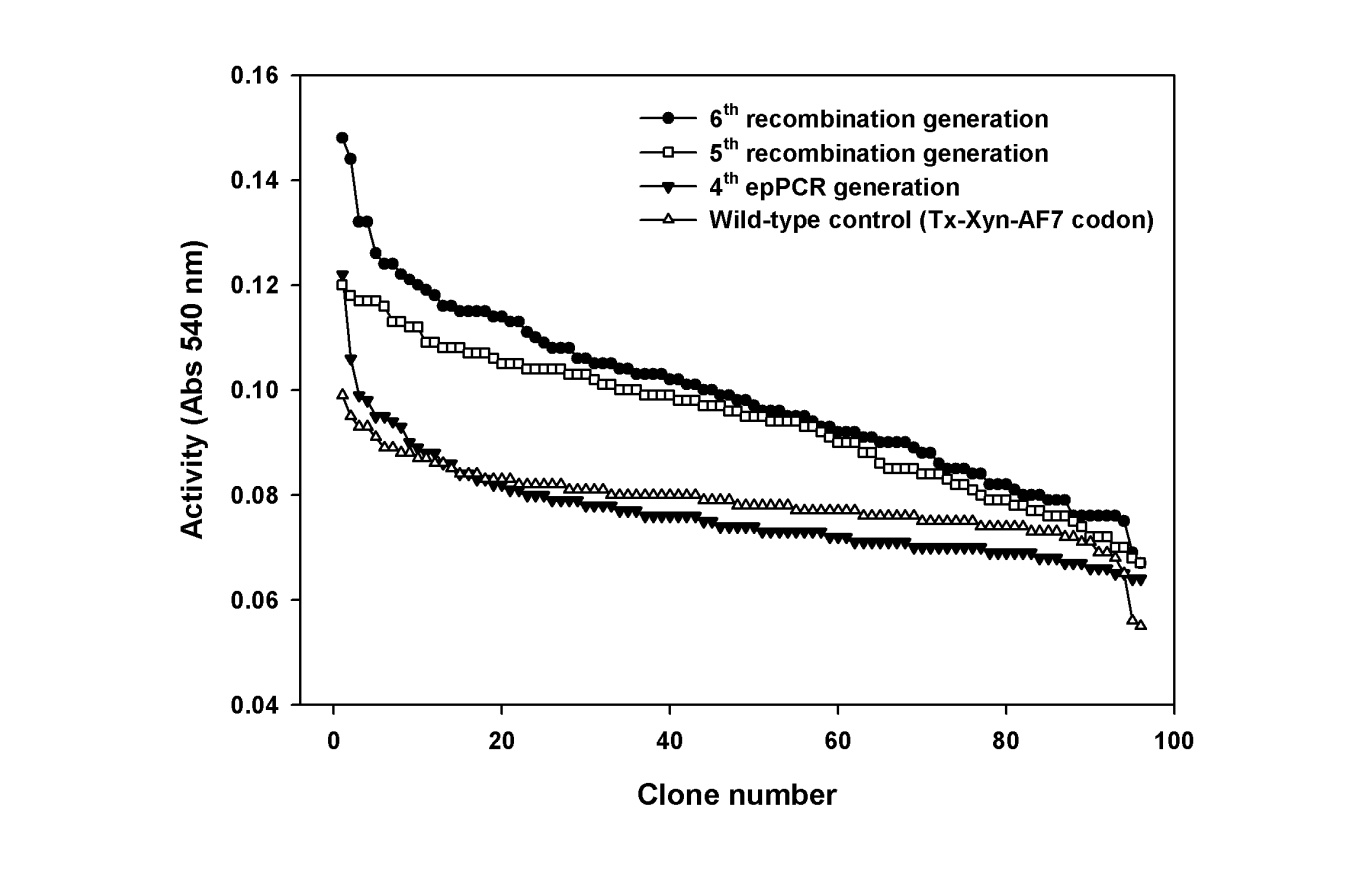
**Iterative improvement of enzyme fitness after screening on xylanase-depleted wheat straw (Dpl-WS)**. The x-axis represents clones in a microtiter plate, randomly selected from a wild-type control series (open triangles, using *Thermobacillus xylanilyticus *xylanase (Tx-Xyn)-AF7 coding sequence), fourth random mutagenesis library (filled triangles), fifth recombinant library (open squares) and sixth recombinant library (filled circles). The y-axis indicates the activity value of corresponding clone in the screening. The same batch of Dpl-WS substrate was used for the four experiments.

To further increment the functional fitness of the enzymes expressed by the candidate clones obtained from random mutagenesis, the staggered extension process (StEP) DNA shuffling approach was adopted, because it offers a much simpler procedure than classical DNA shuffling [29,30]. This method was used to successively create fifth, sixth and seventh-generation libraries. To appreciate the impact of the iterative use of StEP on overall library fitness, Figure 3 shows the relative performance of fourth-generation to sixth-generation libraries. At each generational increment, library fitness increased in accordance with expectations [30-32]. The results of statistical analyses performed on the three successive libraries (fifth, sixth and seventh generations) that were created using this method are summarized in Table 3.

**Table 3:** Mutational frequency in the fifth to seventh generations

Generation	Y6H	Y24F	S27T	S29N	N30D	L64P	W109R	Y111H	K132R	V139A	Y172H
Fifth	22.2%	11.1%	22.2%	11.1%	11.1%	22.2%	11.1%	100%	22.2%	22.2%	11.1%
Sixth	28.6%	14.3%	28.6%	-	14.3%	-	-	100%	14.3%	14.3%	-
Seventh	44.4%	-	55.6%	-	11.1%	-	-	100%	22.2%	22.2%	-

For the initial round of DNA shuffling, 30 clones were used as parental input. After DNA shuffling, the library was submitted to screening using the modified Dpl-WS assay. This step allowed the selection of seven hits whose activities were significantly higher (>7CV) than the mean value of the activity of Tx-Xyn-AF7 clones. DNA sequencing revealed that these 7 clones contained 11 point mutations, including Y111H and some new amino acid substitutions (Figure 2). As before, the seven mutants were used as parental input for two further rounds (sixth and seventh) of DNA shuffling.

After the creation of the seventh-generation library, the experiment was stopped, because DNA sequencing of the highest performing seventh-generation clones showed that five mutational combinations out of a total of seven had already been identified in the sixth generation (Figure 2). This observation suggested that the evolutionary itinerary had almost reached an end, with very little new biodiversity being introduced.

Among the seven best performing seventh-generation clones, Y6H-Y111H and Y6H-S27T-Y111H displayed the highest activity increase (>8 CV) in the screening, compared to that of wild-type control (Tx-Xyn-AF7). In addition, among the six amino acid substitutions that were detected in clones obtained from DNA shuffling, Y111H was present in every template and the frequency of Y6H and S27T increased from the fifth generation to the seventh generation (Table 3). Consequently, we decided to focus on clones containing these three amino acid changes for enzyme production and characterization. Overall mutants that were retained for characterization included Y6H-Y111H, S27T-Y111H and Y6H-S27T-Y111H from the seventh-generation screening and the single mutants Y111H, Y6H and S27T.

### Site-saturation mutagenesis (SSM) at positions 3 and 111

Among the second-generation clones, selected for higher activity on In-WS, two amino acid positions, 3 and 111, were pinpointed as potentially interesting locations. Therefore, in addition to the use of Y3H and Y111H as parental templates for further random mutagenesis and DNA shuffling, SSM was performed to investigate the importance of these two residues with respect to enzyme activity on recalcitrant arabinoxylan (AX) in wheat straw (that is, Dpl-WS). In each case a library was created and 288 clones were screened using the modified Dpl-WS assay. This number of clones was sufficient to ensure a 99.87% probability that all possible amino acid variants were present [33]. Additionally, a random sample of each library was submitted to DNA sequence analysis in order to control the success of the experiment.

**Figure 4:**
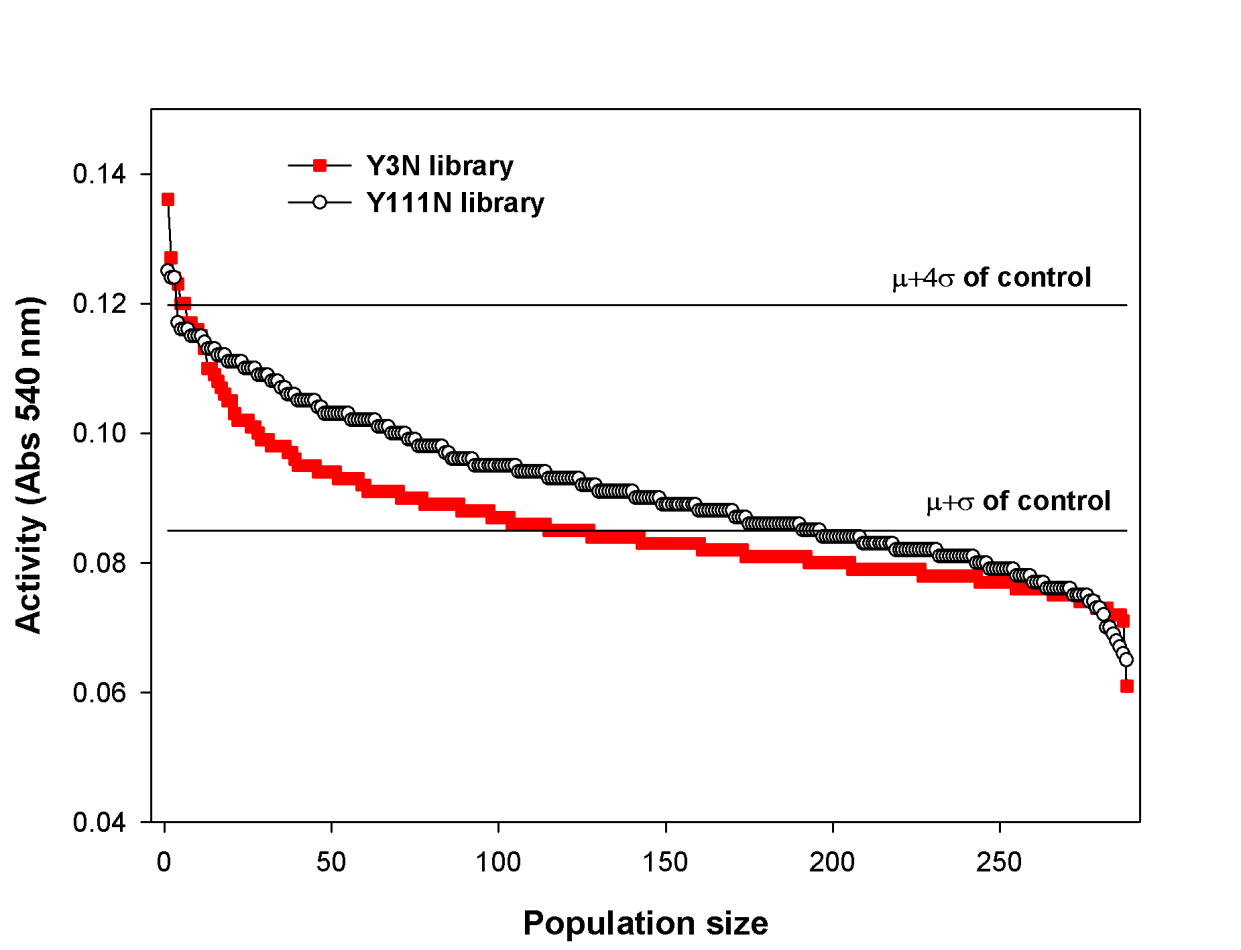
**Xylanase-depleted wheat straw (Dpl-WS) screening of site-saturation libraries**. Filled squares and open circles correspond to site-saturation mutagenesis (SSM) performed at positions 3 and 111, respectively. The 288 clones of each library are positioned on the x-axis, and their activity on Dpl-WS is shown on the y-axis. The two solid lines represent the mean value (μ) and the value of μ + 4σ for the activity of *Thermobacillus xylanilyticus *xylanase (Tx-Xyn)-AF7.

Figure 4 shows the results of the screening of the two site-saturation libraries. Overall, the Y111N (N represents any amino acid) library provides a larger population of improved clones, though both libraries contain a small minority of clones that display activities that are above the value of μ + 4σ of wild-type control (where σ is standard deviation and μ is mean value). Three highest performing clones were selected from each library and analyzed by DNA sequencing. All three clones from the Y3N library displayed the same Y3W mutation, whereas two clones from the Y111N library were phenotypically and genotypically identical (encoding the mutation Y111S) and one displayed an Y111T mutation. In view of these results, three individual clones encoding Y3W, Y111S and Y111T were retained for further characterization.

### Characterization of key properties of the Tx-Xyn mutants

Since the screening of mutant enzyme libraries obeys the maxim 'you get what you screen for', the mutants selected in this work were only improved with respect to the hydrolysis of wheat straw. Hence, other important properties such as thermostability could have been negatively affected. Consequently, the thermostability of each mutant was assessed (Table 4). Although the thermostability of some mutants at 60°C was clearly affected (for example, that of Y6H and Y6H-Y111H), all of the enzymes were sufficiently stable to enable the measurement of kinetic properties without any major modifications to the protocols that were routinely used to characterize wild-type Tx-Xyn. It is also noteworthy that all of the mutants were highly stable at 50°C, since measured activity remained stable over a 6 h incubation period.

**Table 4:** Thermostability of *Thermobacillus xylanilyticus *xylanase (Tx-Xyn) and mutants thereof

Mutant	**T**_**m **_**(°C)**	**t**_**1/2 **_**at 60°C (h)**
Tx-Xyn	75.9	5.4
Y6H	72.9	2.6
S27T	76.4	6.4
Y111H	75.1	3.9
Y6H-Y111H	72.7	2.7
S27T-Y111H	75.4	6.4
Y6H-S27T-Y111H	74.3	3.9
Y3W	73.1	3.2
Y111S	75.1	3.6
Y111T	74.9	5.0

The melting temperature (T_m_) was determined using differential scanning fluorimetry (DSF) and the half-life (t_1/2_) was defined as the period necessary for the initial activity to be reduced by 50% at 60°C.

Each of the mutants was characterized with regard to its ability to hydrolyze birchwood xylan (BWX) and low-viscosity wheat arabinoxylan (LVWAX). According to our findings (data not shown), BWX is devoid of α-L-arabinosyl substitutions, and LVWAX displays an A/X ratio of 0.54. Concerning wild-type Tx-Xyn, its turnover number and performance constant were higher for LVWAX, though the apparent K_M _value was lower on BWX. This tendency was also displayed by the majority of the mutants (Table 5). Regarding the apparent values of K_M_, all of the mutants displayed improved affinity for BWX, but this was not the case for LVWAX. Notably, Y111H was the mutant that displayed the best affinity for BWX, while its affinity for LVWAX was unaltered. However, the rate constant for Y111H-mediated hydrolysis of BWX was lowered when compared to that of the wild-type enzyme, but was improved on LVWAX. Intriguingly, the opposite was true for Y111T, for which the value of *k*_*cat *_was 48% greater than that of Tx-Xyn on BWX, but identical to that of Tx-Xyn on LVWAX. When Y111H was combined with other mutations (for example, S27T-Y111H or Y6H-Y111H), its influence on the performance constant appeared to be dominant, annulling the improved activity on BWX, displayed by the single mutants S27T and Y6H.

**Table 5:** Kinetic parameters of *Thermobacillus xylanilyticus *xylanase (Tx-Xyn) and mutants for hydrolyses involving either birchwood xylan (BWX) or LVWAX

Mutant	**Kinetic parameters**^**a**^						SR^c^
		
	BWX			LVWAX			
		
	*k*_*cat*_(s^-1^)	K_M_^b ^(g/l)	***kcat*/K**_**M**_^**b **^**(s^-1^/g/l)**	*k*_*cat *_(s^-1^)	K_M_^b ^(g/l)	*kcat*/Km^b ^(s^-1^/g/l)	
Tx-Xyn	610.5 ± 19.6	2.54 ± 0.19	242.1	1,699.4 ± 95.9	5.10 ± 0.09	333.1	0.73
Y6H	806.1 ± 61.2	2.37 ± 0.27	340.5	2,081.6 ± 16.4	5.73 ± 0.09	363.0	0.94
S27T	742.9 ± 22.4	1.93 ± 0.15	376.5	1,936.0 ± 19.2	4.81 ± 0.04	402.5	0.94
Y111H	449.3 ± 23.2	1.54 ± 0.14	292.0	1,889.0 ± 72.9	5.01 ± 0.07	376.7	0.78
Y6H-Y111H	433.1 ± 12.5	1.91 ± 0.11	226.8	1,834.7 ± 75.4	5.33 ± 0.35	345.9	0.66
S27T-Y111H	535.8 ± 30.2	1.72 ± 0.21	311.5	1,906.1 ± 4.4	4.39 ± 0.02	434.1	0.72
Y3W	704.5 ± 29.8	2.11 ± 0.14	333.4	1,743.0 ± 26.5	5.51 ± 0.02	316.3	1.05
Y111S	758.8 ± 15.9	2.12 ± 0.04	358.6	1,755.0 ± 106.3	4.75 ± 0.14	369.4	0.97
Y111T	905.2 ± 17.2	2.35 ± 0.15	369.4	1,740.4 ± 41.8	4.81 ± 0.06	361.4	1.02

^a^All data presented are the result of triplicate experiments.

^b^The heterogeneous nature of the substrate excludes the determination of a value for K_M _, thus values are apparent.

^c^Ratio of the performance constants $\left(k_{cat}^{BWX}/K_{M}^{BWX}\right)/\left(k_{cat}^{LVW_{a}X}/K_{M}^{LVWAX}\right)$ for a given enzyme.

### Assessment of the impact of Tx-Xyn mutants on wheat straw

**Figure 5:**
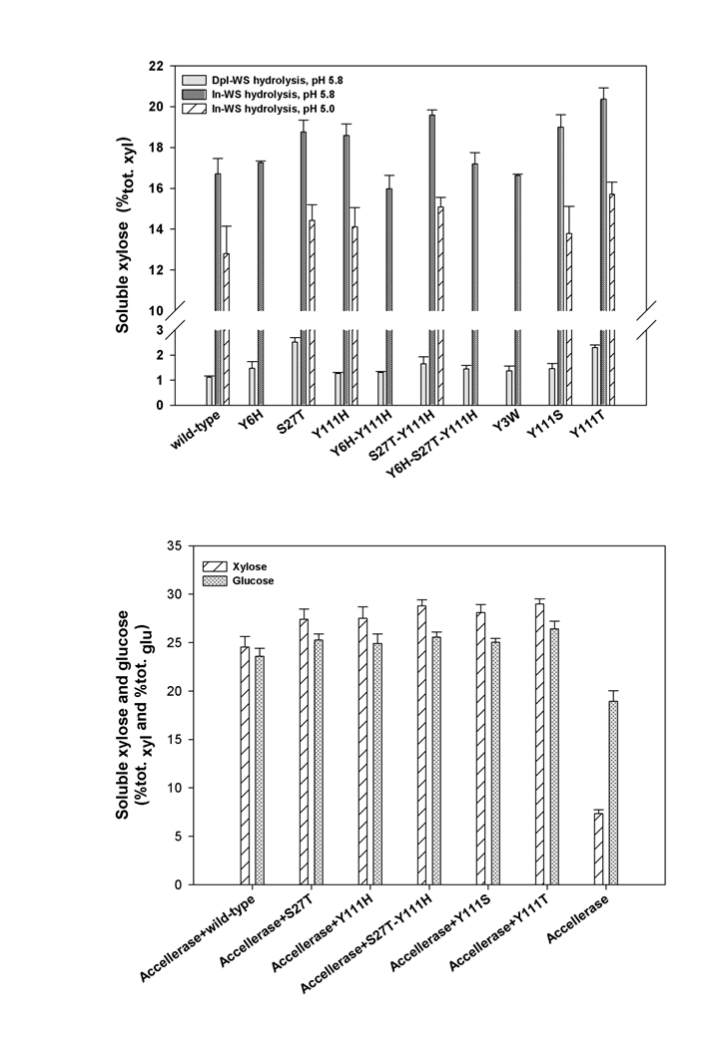
**Percentage conversion of total sugars in xylanase-depleted wheat straw (Dpl-WS) and intact wheat straw (In-WS) by *Thermobacillus xylanilyticus *xylanase (Tx-Xyn) alone or in combination with Accellerase 1500 . (A) **Conversion of total xylose in reactions involving wild-type or mutant Tx-Xyn and Dpl-WS (reactions performed at pH 5.8) or In-WS (pH 5.0 and 5.8) as substrates. **(B) **Conversion of total xylose and glucose in In-WS, using Accellerase 1500 alone or in combination with wild-type and mutant Tx-Xyn (reactions performed at pH 5.0). In both (A) and (B), the x-axis shows the enzyme(s) employed in the corresponding hydrolysis reaction. The method used to derive percentage conversion is described in the Methods section.

To further evaluate the altered properties of the different mutants, their activities on the original wheat straw samples (In-WS and Dpl-WS) were examined. Reactions were performed using pure preparations of wild-type and mutant xylanases either alone or in the combination with Accellerase 1500 (a cellulase cocktail). The results of HPAEC-PAD analyses performed on the reaction supernatants are shown in Figure 5A,B, which show the conversion of total xylose and glucose (that is, %_tot. xyl _and %_tot. glu_, w/w) in the straw residues. The soluble sugar yields are summarized in Additional files 1 and 2.

The hydrolysis of Dpl-WS revealed that all of the mutants could release further amounts of soluble xylose equivalents and that their performance was superior to that of wild-type Tx-Xyn. The mutants S27T and Y111T produced the most outstanding results, because these could release 2.3-fold and 2.1-fold more xylose equivalents from Dpl-WS than Tx-Xyn. The lowest performers were Y111H and Y3W, which yielded 35% and 46% more xylose equivalents, respectively (Figure 5A). However, it should be noted that even the best variant S27T could only release 2.5% _tot. xyl _of Dpl-WS (5.5 g xylose per kg wheat straw), which is evidence of the recalcitrance of this substrate.

For the hydrolysis of In-WS (pH 5.8), wild-type Tx-Xyn released 43.7 g equivalent xylose per kg wheat straw. This represents 4.4% of the dry weight and 16.7% of total xylan (16.7%_tot. xyl_) content. Similar results were obtained for the mutants Y6H, Y6H-Y111H, Y6H-S27T-Y111H and Y3W, but five other mutants yielded higher amounts (18.6% to 20.4%_tot. xyl_) of soluble xylose equivalents, with the best mutant being Y111T (Figure 5A).

The five mutants displaying improved activity on In-WS, were further selected to investigate synergy with cellulases on In-WS, operating at the optimum pH for Accellerase (pH 5.0). Likewise, suitable control reactions at pH 5.0 were performed using only mutant xylanases, or wild-type Tx-Xyn. All controls revealed that the different xylanases displayed reduced hydrolytic capacity, compared to their activity at pH 5.8 (Figure 5A). According to its manufacturer, Accellerase 1500 principally contains endoglucanase and β-glucosidase activities. In our trials, Accellerase alone was able to solubilize 7.3%_tot. xyl _and 18.9%_tot. glu _In-WS (Figure 5B). However, in combination with xylanases, higher yields of xylose and glucose were measured, which were greater than the sum of the yields of Accellerase and xylanase alone, clearly revealing synergistic interactions between the enzyme participants. The mixture of wild-type Tx-Xyn and Accellerase solubilized 24.5%_tot. xyl _and 23.6%_tot. glu _of In-WS (Figure 5B). However, significantly the different mutants were able to improve on this performance, solubilizing 27.4 to 29.0%_tot. xyl _and 24.9 to 26.4%_tot. glu _from In-WS.

## Discussion

### Is enzyme engineering a useful strategy to improve biomass deconstruction?

Artificial enzyme evolution, relying on *in vitro *random mutagenesis and DNA recombination techniques, is a powerful strategy to pinpoint functional determinants and to rapidly improve enzyme fitness with regard to a variety of physical or biochemical properties [34-36]. However, the need for an appropriate screen is vital. In this work, we relied on a previously described screening method, which allowed us to address a highly ambitious target, which was the isolation of enzymes that display higher activity on raw biomass. To our knowledge, no such enzyme engineering has yet been attempted, mainly because biomass-degrading enzymes are improved for their activity on artificially isolated biodiversities or pretreated biomass, wherein the notion of chemical and structural complexity is totally omitted or mainly cellulose is present, with lignin and hemicelluloses being very minor components [37-39].

Therefore, the underlying rationale of our approach was to investigate to what extent the fitness of a xylanase, or for that matter any other biomass-degrading enzyme, can be independently improved for hydrolysis of complex biomass, without interfering with the structural and chemical complexity of the substrate. Likewise, we hoped to provide a novel angle on the understanding of the factors that govern the enzymatic deconstruction of raw biomass.

Our previous study revealed that the Tx-Xyn-mediated hydrolysis of wheat straw is a complex reaction that cannot be modeled using Michaelis-Menten kinetics and does not reach completion even at high enzyme loading and after long time periods [24,28]. To achieve the first phase of the reaction requires quite long incubation times (approximately 8 h), thus screening using raw wheat straw (that is, In-WS) provides a means to find variants that display improved initial catalytic rates, which can result either from the improvement of intrinsic catalytic properties of the xylanase, or from an increase in enzyme production. However, the use of In-WS is not appropriate to isolate xylanases that will surpass the sugar solubilization yield of the wild-type Tx-Xyn. For this purpose, it is more appropriate to use Tx-Xyn-pretreated wheat straw (that is, Dpl-WS), which should provide a means to identify enzyme variants that can accelerate the latter phase of the reaction and better surmount the obstacles that prevent further action by Tx-Xyn. Therefore, in the strategy developed here, both screening approaches were applied, first in an attempt to accelerate the reaction and second to improve the overall impact of xylanase action on wheat straw.

Overall, all of the qualitative indicators that are presented here show that the enzyme evolution approach was successful in increasing the fitness of Tx-Xyn for biomass hydrolysis. At each step, clones with ever increasing activity could be selected and the ultimate analysis of the best clones revealed that several could actually better hydrolyze wheat straw, especially when their action was coupled to a cellulose cocktail. Nevertheless, unsurprisingly the overall impact of the improvements was modest, but these results need to be considered in the light of current knowledge.

Two recent studies [3,40] have attempted to relate enzyme action on wheat straw to changes at the ultrastructural level. These authors have shown that a mild hydrothermal pretreatment (185°C, 10 min) releases approximately 34% of available xylans (that is, approximately 8.2% of the initial DW), which appear to come from the pith that lines the central lumen of wheat straw. Further treatment of the sample with a cellulase cocktail released glucose and xylose from cellulose microfibrils and xylans, respectively, apparently present in the parenchyma cells that form the cortex. However, enzymatic degradation was impotent on lignified cells (for example, sclerenchyma cells). In our experiments, total xylans in wheat straw represent approximately 26% DW and Tx-Xyn can release 16.7% of these (that is, 4.4% DW). The mutant Y111T is able to solubilize approximately 21.9%_tot. xyl _or 5.3% DW over a 24-h period. Taken together, our results reveal that the hydrolysis of wheat straw using Tx-Xyn variants procures solubilization yields that are inferior, but not dissimilar, to those obtained using mild hydrothermal treatment, and thus it is tempting to suggest Tx-Xyn also preferentially hydrolyzes pith and parenchyma cells.

The failure of Tx-Xyn, or variants thereof, to further solubilize xylans is probably not linked to intrinsic catalytic potency or to substrate selectivity of Tx-Xyn and its mutants, but rather to the inaccessibility of the substrate. Indeed, coupling of wild-type Tx-Xyn to that of a cellulase cocktail clearly revealed a certain degree of synergy, releasing approximately 24% of the theoretical yield of sugars. Significantly, mutants generated in this work amplified this synergy and achieved higher levels of sugar solubilization, indicating that the enzymatic removal of cellulose exposes xylan and vice versa. Possibly, the improved action of the mutants allows a slightly more profound degradation of the parenchyma cells that form the cortex of wheat straw. However, the results of this study indicate that enzyme engineering alone cannot overcome the limits imposed by the lignin barrier, which is progressively exposed by the peeling action of the xylanase/cellulases cocktail.

### Structure-function relationships revealed in this study

**Figure 6:**
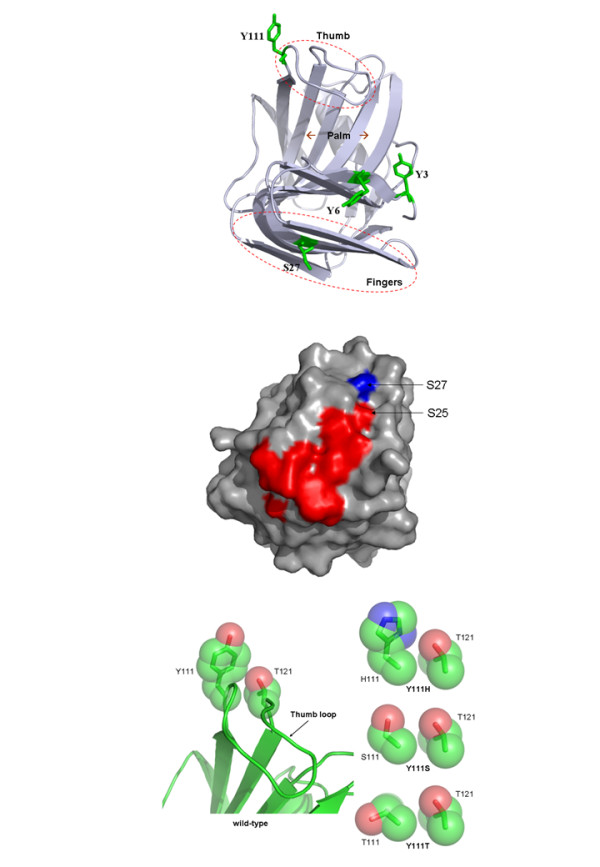
**Localization of key mutations in the three-dimensional structure of *Thermobacillus xylanilyticus *xylanase (Tx-Xyn) . (A) **Positions of Y3, Y6, S27 and Y111 residues. **(B) **Potential secondary binding site determinants (red), including S27 (blue), mutated in this study. **(C) **Space-filling model showing the spatial occupation of side chains at positions 111 and 121 in Tx-Xyn and in the mutants Y111H, Y111S and Y111T. All figures were prepared using PyMol software [64].

One of the remarkable findings in this study is the identification of a relatively small number of mutations. After six rounds of combined mutagenesis and DNA shuffling, seven mutants possessing a total of six point mutations were identified. Among these mutations, three emerged (amino acids 6, 27 and 111) as important positions, because of their reoccurrence in the seven mutants. In addition, another three mutants (Y3W, Y111S and Y111T) were isolated from SSM libraries, in which amino acids 3 and 111, respectively were targeted. Tyr3 and Tyr6 are located at the B2 β strand in the N-terminal region of Tx-Xyn, whereas Ser27 forms part of the 'knuckles' region of fingers and Tyr111 is located on the thumb (Figure 6A). The examination of the different combinations that were obtained reveals that generally these mutations did not provide additive benefits. For example, regarding the mutants Y6H-Y111H, S27T-Y111H and Y6H-S27T-Y111H, the two point mutation variants Y6H and S27T displayed greater hydrolytic potency on Dpl-WS than any of these combinations. Similarly, S27T displayed the highest catalytic efficiency towards the two soluble xylan substrates, BWX and LVWAX. Therefore, it appears legitimate to consider the impacts of the different mutations independently.

The findings presented here concerning the reduced thermostability of mutants displaying substitutions at positions 6 (Y6H) and/or 111 (Y111H) clearly provide support for the existence of hydrophobic patches that might mediate the oligomerization, and thus the thermostabilization, of Tx-Xyn in solution. According to Harris *et al*. [41], Tyr6 and Tyr111 are surface exposed aromatic amino acids that along with nine other aromatic residues participate in the formation of intermolecular 'sticky patches' that form the basis for thermostability in Tx-Xyn. Nevertheless, it is also important to note that not all mutations at position 111 produced the same effect. Notably, the mutant Y111T displayed thermostability very close to that of the wild-type Tx-Xyn. Interestingly, the mutant S27T actually increased thermostability, which agrees with a trend among certain proteins, including GH11 xylanases, that correlates thermostability with an increased Thr:Ser ratio [42,43].

Among the six mutants bearing single substitutions, S27T, Y111H, Y111S and Y111T displayed improved hydrolysis of In-WS and synergy with the cellulase cocktail. However, the selection of the mutants Y6H and Y3W in our assay was more surprising, because these single mutants did not appear to improve wheat straw hydrolysis, although their specificity towards BWX was clearly altered and Y6H displayed the highest *k*_cat _value on both BWX and LVWAX. The mutants S27T, Y111S and Y111T also showed increased specificity towards BWX, indicating that all single site mutants selected in our assay had acquired an improved ability to hydrolyze less substituted xylans, displaying an Ara:Xyl ratio that is comparable to that of wheat straw xylan (Ara:Xyl ratio of 0.091). Curiously, the only exception to this trend was the double mutant Y6H-Y111H, which displayed unaltered specificity on In-WS, when compared to wild-type Tx-Xyn.

The amino acid Ser27 is located in a region that has been identified as a secondary binding site (SBS) in the GH11 xylanases from *Bacillus circulans *[14] and *Bacillus subtilis *[44,45]. Tx-Xyn shares 73% amino acid identity with the xylanase from *B. circulans *xylanase, and this figure increases to 81% when one just considers the SBS determinants, suggesting that a functional SBS might be present in Tx-Xyn (Figure 6B). In this context, it is noteworthy that Ser27 is located in a relatively deep part of a surface groove in Tx-Xyn that is linked to a shallower region via Ser25, and that surface grooves are potential ligand binding sites [46]. Therefore, one can speculate that Ser27 constitutes an element of a SBS in Tx-Xyn. Functionally, it is proposed that the SBS in certain GH11 xylanases interacts with three or four xylosyl units via hydrogen bonds and Van der Waals interactions, and possibly improves binding of xylan polymers in the active site cleft [14]. The mutation of Ser27 to Thr certainly leads to a localized increase in hydrophobicity, which is probably favorable for xylan binding to the putative SBS. Indeed, experimental evidence supports this, because the mutant S27T significantly reduced the Michaelis constant for the hydrolysis of BWX and, to a lesser extent, for LVWAX. In this respect, it is also noteworthy that among the other mutations identified during the directed evolution process (Table 3), figure S29N, N30D and V139A, which are also in the vicinity of the putative SBS region in Tx-Xyn. Therefore, a complementary study of these mutations could be an interesting way forward to better define the Tx-Xyn SBS and understand its effect on the enzyme activity.

The thumb loop is known to be of prime functional importance in GH11 xylanases. The open and closing of this loop almost certainly plays a key role in substrate selectivity, binding [47-49] and product release [50]. Regarding substrate binding, the conserved tip of the thumb, composed of the motif Pro-Ser-Ile (position 114 to 116 in Tx-Xyn), is involved in binding of xylosyl residues at the -1 and -2 subsites via hydrogen bonds [45,51,52]. Tyr111 and its opposing neighbor Thr121 are located at the base of the loop where they control the movement of this structure [50,53]. The mutation of Tyr111 to either His, Ser or Thr reduces the spatial occupancy at position 111 (Figure 6C), although this is less so for His, and thus probably renders the loop more mobile and more inclined to fold downwards and inwards towards the -1 and -2 subsites. The overall effects of these changes would be improved catalytic turnover and possibly improved binding affinity, both of which are observed for the mutants Y111S and Y111T.

Regarding the loop movement, the mutation of Tyr6 is also worth considering. The relatively conservative substitution of this residue by a slightly less bulky histidine clearly improved the enzyme turnover on both BWX and LVWAX, but had a slightly negative effect on substrate affinity in the case of LVWAX. This implies that Tyr6 might influence the movement of the loop, although a direct interaction is impossible. Nevertheless, Trp7 forms part of the -2 subsite and faces Pro114 and Ile116, which form the thumb tip. Slight adjustments in the position of Trp7 could facilitate the open-close movement of the thumb loop, with the risk of disturbing the high-energy interaction between this residue and the -2 xylosyl moiety.

Finally it is noteworthy that many of the mutations that were identified in this study involved the loss of aromatic side chains. Often, the non-productive binding by lignin is cited as a major cause of enzyme inefficiency on lignocellulosic biomass [54-57]. In an earlier study, it was shown that wild-type Tx-Xyn was strongly absorbed by both wheat straw and isolated wheat straw lignin [28]. In a more recent study [58], it has been shown that phenolic acids can act as non-competitive multisite inhibitors of Tx-Xyn that might provoke conformational alterations of the enzyme. Therefore, it is tempting to speculate that the elimination of surface exposed aromatic amino acid side chains might lower such inhibitory effects.

## Conclusions

Using a random mutagenesis and directed evolution approach we have been able to generate a number of mutants whose behavior is globally coherent with the screening assay that was employed. Several mutants display improved hydrolytic activity on wheat straw and show increased synergy with cellulase, though none are sufficiently potent to be able to overcome the accessibility barrier, which inevitably blocks the way to further hydrolysis of polysaccharides.

## Methods

### General materials and regents

Unless otherwise stated, all chemicals were of analytical grade and purchased from Sigma-Aldrich (St Louis, MO, USA). The T7-promoter based vector pRSETa was purchased from Invitrogen (Cergy Pontoise, France), and the *Escherichia coli *host strains Novablue(DE3) and JM109(DE3) were obtained from Stratagene (La Jolla, CA, USA) and Novagene (Darmstadt, Germany), respectively. All restriction enzymes, T4 DNA ligase, *Taq *DNA polymerase and their corresponding buffers were purchased from New England Biolabs (Beverly, MA, USA). Oligonucleotide primers were synthesized by Eurogentec (Angers, France), and the DNA sequencing was performed by GATC Inc. (Marseille, France). Sterile 96-well cell culture microtiter plates and sealing tapes were purchased from Corning Corp. (NY, USA), and other polypropylene microtiter plates were from Evergreen Scientific (Los Angeles, CA, USA). The low viscosity wheat flour arabinoxylan (LVWAX) was obtained from Megazyme (Wicklow, Ireland), and the birchwood xylan (BWX) was purchased from Sigma-Aldrich.

### Mutagenesis procedure and library construction

Random mutagenesis was carried out by epPCR using an established protocol [31]. The template was (first round only) the DNA encoding Tx-Xyn (Swiss-Prot accession number Q14RS0, bearing the substitution N1A) or (in subsequent rounds) Tx-Xyn-AF7 described by Song *et al*. [24]. Briefly, the PCR reaction mixture (50 μl) contained 5 ng of template DNA, 0.3 μM of primers epF and epR (see below), 0.2 mM dGTP/ATP (equimolar mixture) and 1 mM dCTP/TTP (equimolar mixture), 7 mM MgCl_2_, 5 IU *Taq *polymerase and (in the third round of epPCR only) 0.05 mM of MnSO_4_. Reactions were conducted using the following sequence: 1 cycle at 94°C for 2 min, 30 cycles at 94°C for 1 min, 1 cycle at 42°C for 1 min and 1 cycle at 72°C for 1 min, and finally 1 cycle at 72°C for 5 min. The amplicons were purified using QIAquick PCR Purification Kit (Qiagen, Courtaboeuf, France) and were digested with *Eco*RI and *Nde*I and inserted into a similarly digested pRSETa vector. The ligation mixture was used to transform competent *E. coli *Novablue (DE3) cells. epF: 5'-GGAGATATACAT**ATG**GCCACG-3'; epR: 5'-GGATCAAGCTTCGAATTCTTACC-3'. DNA recombination was carried out using an adapted StEP method [30,32]. The PCR reaction (50 μl) contained 5 ng of total template DNA (equimolar mixture of each parental gene), 0.3 μM of each primer, 0.2 mM of each dNTP, and 5 IU *Taq *polymerase. Reactions were conducted using the following sequence: 1 cycle at 94°C for 2 min; 40 cycles comprising a step at 94°C for 30 s and 1 step at 58°C for 2 s; followed by 40 cycles with 1 step at 94°C for 30 s and 1 step at 56°C for 2 s. Afterwards, 20 IU of *Dpn*I was added to the PCR reaction, which was incubated at 37°C for 1 h, before amplicon purification and digestion with *Eco*RI and *Nde*I. Finally, the mutant library was generated by ligating the digested amplicons to *Eco*RI/*Nde*I-digested pRSET plasmid DNA and transforming the resultant products into competent *E. coli *Novablue(DE3) cells.

Site-saturation mutagenesis on residues Tyr3 and Tyr111 of Tx-Xyn was performed using the QuikChange mutagenesis kit (Stratagene, La Jolla, CA). The following mutagenic primers (Eurogentec) were designed using NNK degeneracy [59], according to the recommendations provided in the instruction manual (mismatched bases are underlined; N is A, G, C, or T; K is G or T; M is A or C). For amino acid position 3: Y3N_FW: 5'-GATATACATATGGCCACG**NNK**TGGCAGTATTGGACG-3'; Y3N_REV: 5'-CGTCCAATACTGCCA**MNN**CGTGGCCATATGTATATC-3'. For amino acid position 111: Y111N_FW: 5'-C TATCACAGCTGGCGC**NNK**AACGCACCGTCC ATCGAC-3'; Y111N_REV: 5'-GTCGATGGACGGTGCGTT**MNN**GCGCCAGCTGTGATAG-3'. Following PCR, a digestion with *Dpn*I removed template DNA, and the product was used to transform *E. coli *Novablue (DE3) cells.

The mutational combinations W109R-Y111H, Y3H-W109R-Y111H, Y3L-W109R-Y111H, S27T, and Y6H were created through site-directed mutagenesis. This was achieved using the QuikChange site-directed mutagenesis kit, according to the manufacturer's instruction. The oligonucleotide primers employed in PCRs are listed in Additional file 3.

### Library screening on intact and xylanase treated wheat straw

Wheat straw (*Triticum aestivum*, cv. Apache) harvested (2007) in France was milled using a blade grinder that procured a fine powder having an average particle size of 0.5 mm. After, the wheat straw powder, designated In-WS, was washed with distilled water (10 volumes), filtered using a Büchner funnel equipped with Whatman No.4 filter paper (pore size: 20 to 25 μm), dried in an oven at 45°C and then sterilized by autoclaving. To prepare xylanase-treated wheat straw (designated Dpl-WS), 20 g In-WS were suspended in 50 mM sodium acetate buffer, pH 5.8 (containing 0.02% NaN_3_) containing Tx-Xyn (150 BWX U/g biomass) and incubated at 60°C for 70 h. Afterwards, the reaction mixture was heated at 95°C for 5 min to inactivate the enzyme. The solid residues were recovered by filtration (see above) and dried as before. The sugar composition of both wheat straw substrates (Table 2) was analyzed according an established protocol [60].

Microtiter plate-based screening of mutant libraries was performed according to the method described by [24]. Briefly, individual *E. coli *transformants were grown in the wells of 96-well microtiter plates and then cells were recovered and lysed using the combined effect of lysozyme (0.5 g/l) and freeze-thaw cycling (-80°C and 37°C). The screening of xylanase activity was then achieved using a four-step protocol, which involved (1) substrate delivery into microtiter plates (2) addition of xylanase-containing cell lysates (3) incubation and (4) measurement of solubilized reducing sugar using a micro-DNS assay. The important experimental details of these steps are summarized in Table 1. When Dpl-WS was employed in the place of In-WS, the incubation time was extended to 16 h and, consequently, microtiter plates were thermosealed using polypropylene film to reduce evaporation. In all microtiter plate screening, wells containing transformants expressing wild-type Tx-Xyn were included as internal controls. These were used to calculate a coefficient of variation (1 CV = σ/μ × 100%) of Tx-Xyn activity, which was employed to assess the activity of mutant variants.

### Xylanase expression and purification

The production in *E. coli *JM109(DE3) cells and purification of Tx-Xyn and variants thereof was performed according to the previously described procedure [61]. Briefly, purification followed a two-step protocol involving ion-exchange (Q sepharose FF) and then affinity chromatography (Phenyl sepharose) operating on an ÄKTA purification system (GE Healthcare, Uppsala, Sweden). Enzyme conformity and purity were assessed using SDS-PAGE and theoretical extinction coefficients were computed using the ProtParam server [62]. The concentration of xylanase solutions was determined by measuring UV absorbance at 280 nm and then applying the Lambert-Beer equation.

### Evaluation of xylanase-mediated hydrolysis on Dpl-WS and In-WS

To measure xylanase activity using In-WS or Dpl-WS as substrates, a reaction mixture in 50 mM sodium acetate buffer, pH 5.8 was prepared that contained 2% (w/v) biomass, 0.1% (w/v) bovine serum albumin (BSA), 0.02% (w/v) NaN_3 _and an aliquot (final concentration of 10 nmol enzyme/g biomass) of Tx-Xyn or a mutant thereof. To analyze the combined effect of xylanase and cellulase on In-WS, reactions were conducted as described above, except that Accellerase 1500 (Genencor, Rochester, NY, USA) (0.2 ml cocktail per g biomass) was added to the reaction mixture and reactions were buffered at pH 5.0. To assess the action of Accellerase 1500 alone, xylanase was omitted.

All hydrolyses were performed at 50°C for 24 h with continuous stirring (250 rpm) in screw-capped glass tubes, and then stopped by heating at 95°C for 5 min. For analysis, the reaction mixture was centrifuged (10,000 *g *for 2 min) and then the supernatant was filtered (polytetrafluoroethylene, 0.22 μm), before injection onto a high performance anion exchange chromatography system with pulsed amperometric detection (HPAEC-PAD). For monosaccharide analysis, separation was achieved at 30°C over 25 min on a Dionex CarboPac PA-1 column (4 × 200 mm), equipped with its corresponding guard column and equilibrated in 4.5 mM NaOH and running at a flow rate of 1 ml/min. For the analysis of xylo-oligosaccharides (XOS), a Dionex CarboPac PA-100 column (4 × 200 mm), equipped with its corresponding guard column and equilibrated in 4.5 mM NaOH was employed. Separation of various XOS was achieved by applying a gradient of NaOAc (5 to 85 mM) in 150 mM NaOH over 30 min at 30°C, using a flow rate of 1 ml/min. Appropriate standards (monosaccharides such as L-arabinose, D-xylose, D-glucose and D-galactose and various XOS displaying a degree of polymerization of 2 to 6) at various concentrations (2 to 25 mg/l) were used to provide quantitative analyses. Finally, the quantitative results from HPAEC analysis (monomeric and oligomeric sugars) were converted into the amount of soluble monosaccharide equivalents (designated 'average solubilized weight'), and the percentage conversion was calculated as follows, either in terms of xylose or glucose:

$$\text{Conversion}\;{\%}_{tot.N}=\frac{\text{average}\;\text{solubilized}\;N}{\text{theoretical}\;\text{total}\;N}\times 100\%\;\left(w/w\right)$$

Where '*N*' represents xylose or glucose, and the 'theoretical total *N' *is the total amount of sugar *N *present in the initial straw sample (Table 1).

### Determination of kinetic parameters

To measure the kinetic parameters of Tx-Xyn and its mutants, BWX and LVWAX were used as substrates at eight different concentrations (0 to 12 g/l). Hydrolysis reactions (1 ml) were performed at 60°C in NaOAc, pH 5.8 using approximately 4.5 and 3.5 nM of xylanase for BWX and LVWAX assays, respectively. During the course of the reaction, aliquots (100 μl) were removed at 3-min intervals, and immediately mixed with an equal volume of 3,5-dinitrosalicylic acid (DNS) reagent to stop the reaction. The quantity of solubilized reducing sugars present in samples was assessed by the DNS assay [63]. Finally, results were analyzed using SigmaPlot V10.0, which generated values for *k*_cat _and K_M_. Taking into account the heterogeneous nature of the substrates, computed K_M _values are apparent values having units of g/l.

### Thermostability assay

To measure the thermostability of the xylanases used in this study, enzyme solutions (100 mM in 10 mM Tris-HCl buffer, pH 8.0) were incubated at 50°C and 60°C for up to 6 h. At intervals, aliquots were removed and used to measure residual xylanase activity on BWX (at 5 g/l) at 60°C using the DNS method to quantify solubilized reducing sugars. One unit (1 U BWX) of xylanase activity was defined as the amount of xylanase required to release 1 μmol of equivalent xylose per minute from BWX. Enzyme half-life (t_1/2_) was deduced by fitting the curve of ln(residual activity) = *k*t where t is the time and *k *is the slope, and t_1/*2 *_*= k *^-1^ln(0.5) [16].

### Determination of melting temperature by differential scanning fluorimetry (DSF)

A sample in 20 mM Tris-HCl buffer, pH 8.0 was prepared that contained 100 mM NaCl, SYPRO Orange (Invitrogen, final concentration 10 ×), and an aliquot (final concentration of 6.75 μM) of Tx-Xyn or mutant xylanases thereof. Negative controls containing either SYPRO or xylanase alone were analyzed in parallel. A CFX96 Real-Time PCR Detection System (Bio-Rad) was used as a thermal cycler and the fluorescence emission was detected using the Texas Red channel (λ_exc _= 560 to 590 nm, λ_em _= 675 to 690 nm). The PCR plate containing the test samples (20 μl per well) was subjected to a temperature range from 20°C to 99.5°C with increments of 0.3°C every 3 s. The apparent melting temperature (T_m_) was calculated by the Bio-Rad CFX Manager software.

## Competing interests

The authors declare that they have no competing interests.

## Authors' contributions

LS performed this work as part of his doctoral studies, and thus performed the majority of the work, participated in data analysis and wrote the first draft of the manuscript. MOD was the principal investigator and thesis director, responsible for the study design, analysis of the results and cowriting of the manuscript. SB coordinated the experimental work and performed some of the site-saturation mutagenesis work. CD helped with enzyme kinetics and data analysis and BS performed the T_m _measurements. SB, CD and BS all corrected the manuscript and approved the final version.

## Supplementary Material

Additional file 1 **Equivalent xylose yields from hydrolyses involving Tx-Xyn and mutants**. Click here for file

Additional file 2 **Equivalent xylose and glucose yields (recorded at 24 h) from the hydrolysis of In-WS by a mixture of Accellerase 1500 and Tx-Xyn or mutants thereof**. Click here for file

Additional file 3 **Oligonucleotide primer pairs used for site-directed mutagenesis**. Click here for file
