# Supplementary material for: Engineering better biomass-degrading ability into a GH11 xylanase using a directed evolution strategy
Source: Biotechnol Biofuels. 2012 Jan 13;5:3. doi: 10.1186/1754-6834-5-3 (PMC3299623; doi:10.1186/1754-6834-5-3)
Supplement: Additional file 1 — Equivalent xylose yields from hydrolyses involving Tx-Xyn and mutants. [file 1754-6834-5-3-S1.DOC]

**Additional file 1 Equivalent xylose yields from hydrolyses involving Tx-Xyn and mutants.** Measurements were recorded after 24h and reactions containing Dpl-WS were performed at pH 5.8, while those containing In-WS were performed both at pH 5.0 and 5.8.

| Equivalent xylose yields (g. kg^-1^ biomass) | | | | | | | | |
| --- | --- | --- | --- | --- | --- | --- | --- | --- |
| Xylanase | Dpl-WS, pH 5.8 | |  | In-WS | | | | |
|  |  |  |  | pH 5.8 | |  | pH 5.0 | |
|  | µ | σ |  | µ | σ |  | µ | σ |
| wild-type | 2.5 | 0.1 |  | 43.7 | 2.1 |  | 33.5 | 4.6 |
| Y6H | 3.2 | 0.6 |  | 45.1 | 0.4 |  | ND | |
| S27T | 5.5 | 0.4 |  | 49.1 | 1.5 |  | 37.8 | 2.0 |
| Y111H | 2.8 | 0.1 |  | 48.6 | 1.5 |  | 36.9 | 2.5 |
| Y6H-Y111H | 2.9 | 0.1 |  | 41.8 | 1.7 |  | ND | |
| S27T-Y111H | 3.6 | 0.6 |  | 51.3 | 1.2 |  | 39.5 | 1.2 |
| Y6H-S27T-Y111H | 3.2 | 0.3 |  | 45.0 | 1.4 |  | ND | |
| Y3W | 3.0 | 0.4 |  | 43.5 | 0.2 |  | ND | |
| Y111S | 3.2 | 0.5 |  | 49.7 | 2.2 |  | 36.1 | 3.4 |
| Y111T | 5.0 | 0.2 |  | 53.3 | 1.4 |  | 41.1 | 1.6 |
| µ and σ: mean value and standard deviation of triplicate measurements; ND: not determined | | | | | | | | |
