# Supplementary material for: Engineering better biomass-degrading ability into a GH11 xylanase using a directed evolution strategy
Source: Biotechnol Biofuels. 2012 Jan 13;5:3. doi: 10.1186/1754-6834-5-3 (PMC3299623; doi:10.1186/1754-6834-5-3)
Supplement: Additional file 2 — Equivalent xylose and glucose yields (recorded at 24 h) from the hydrolysis of In-WS by a mixture of Accellerase 1500 and Tx-Xyn or mutants thereof. [file 1754-6834-5-3-S2.DOC]

**Additional file 2** Equivalent xylose and glucose yields (recorded at 24 h) from the hydrolysis of In-WS by a mixture of Accellerase 1500 and Tx-Xyn or mutants thereof.

| Enzyme | xylose(g. kg^-1^ biomass) | |  | glucose(g. kg^-1^ biomass) | |
| --- | --- | --- | --- | --- | --- |
|  | µ | σ |  | µ | σ |
| wild-type+Accellerase | 64.2 | 2.9 |  | 105.0 | 3.6 |
| S27T+Accellerase | 71.7 | 2.7 |  | 112.4 | 2.8 |
| Y111H+Accellerase | 72.0 | 3.0 |  | 110.8 | 4.3 |
| S27T-Y111H+Accellerase | 75.3 | 1.7 |  | 113.7 | 2.4 |
| Y111S+Accellerase | 73.5 | 2.1 |  | 111.4 | 1.8 |
| Y111T+Accellerase | 75.8 | 1.4 |  | 117.5 | 3.6 |
| Accellerase | 19.2 | 1.0 |  | 84.3 | 4.9 |
|  |  |  |  |  |  |
| µ and σ: mean value and standard deviation of triplicate measurements | | | | | |
