# Supplementary material for: Engineering better biomass-degrading ability into a GH11 xylanase using a directed evolution strategy
Source: Biotechnol Biofuels. 2012 Jan 13;5:3. doi: 10.1186/1754-6834-5-3 (PMC3299623; doi:10.1186/1754-6834-5-3)
Supplement: Additional file 3 — Oligonucleotide primer pairs used for site-directed mutagenesis. [file 1754-6834-5-3-S3.DOC]

**Additional file 3 Oligonucleotide primer pairs used for site-directed mutagenesis.**

| Mutation | Primer sequence (5' 🡪 3') |
| --- | --- |
| W109R and Y111H | CTATCACAGCCGGCGCCACAACGCACCGTCCATC GATGGACGGTGCGTTGTGGCGCCGGCTGTGATAG |
| Y6H | CCACGTACTGGCAGCATTGGACGGACGGC GCCGTCCGTCCAATGCTGCCAGTACGTGG |
| S27T | GCAACTACAGCGTAACCTGGAGCAACAGCGG CCGCTGTTGCTCCAGGTTACGCTGTAGTTGC |
